# Emotion and Deliberative Reasoning in Moral Judgment

**DOI:** 10.3389/fpsyg.2012.00328

**Published:** 2012-09-05

**Authors:** Denise Dellarosa Cummins, Robert C. Cummins

**Affiliations:** ^1^Department of Psychology, University of Illinois at Urbana-ChampaignChampaign, IL, USA; ^2^Department of Philosophy, University of Illinois at Urbana-ChampaignUrbana, IL, USA

**Keywords:** moral decision-making, moral judgment, dual process, emotion

## Abstract

According to an influential dual-process model, a moral judgment is the outcome of a rapid, affect-laden process and a slower, deliberative process. If these outputs conflict, decision time is increased in order to resolve the conflict. Violations of deontological principles proscribing the use of personal force to inflict intentional harm are presumed to elicit negative affect which biases judgments early in the decision-making process. This model was tested in three experiments. Moral dilemmas were classified using (a) decision time and consensus as measures of system conflict and (b) the aforementioned deontological criteria. In Experiment 1, decision time was either unlimited or reduced. The dilemmas asked whether it was appropriate to take a morally questionable action to produce a “greater good” outcome. Limiting decision time reduced the proportion of utilitarian (“yes”) decisions, but contrary to the model’s predictions, (a) vignettes that involved more deontological violations logged faster decision times, and (b) violation of deontological principles was not predictive of decisional conflict profiles. Experiment 2 ruled out the possibility that time pressure simply makes people more like to say “no.” Participants made a first decision under time constraints and a second decision under no time constraints. One group was asked whether it was appropriate to take the morally questionable action while a second group was asked whether it was appropriate to refuse to take the action. The results replicated that of Experiment 1 regardless of whether “yes” or “no” constituted a utilitarian decision. In Experiment 3, participants rated the pleasantness of positive visual stimuli prior to making a decision. Contrary to the model’s predictions, the number of deontological decisions increased in the positive affect rating group compared to a group that engaged in a cognitive task or a control group that engaged in neither task. These results are consistent with the view that early moral judgments are influenced by affect. But they are inconsistent with the view that (a) violation of deontological principles are predictive of differences in early, affect-based judgment or that (b) engaging in tasks that are inconsistent with the negative emotional responses elicited by such violations diminishes their impact.

## Introduction

A belief dating back to ancient times is that emotions cloud good judgment. This adage implies a common sense belief that we have two competing systems for making decisions, one based on emotion and one based on reason. Recently, Greene (2007) proposed a dual-process model which explains moral judgment as the outcome of neurologically separable affective and deliberative processes. According to this model, strong affective responses are the domain of the ventromedial prefrontal cortex (VMPC). Deliberative decision-making is subserved by the dorsolateral prefrontal cortex. When there is no prepotent affective response, deliberative reasoning prevails. When a conflict between the outputs of these areas occurs, it is detected by the anterior cingulate cortex, which signals the need for cognitive control. This need is answered by the anterior dorsolateral prefrontal cortex; if the latter prevails, the output of the deliberative system is selected. If not, the prepotent affective response constitutes the judgment.

This dual-process explanation of moral judgment is consistent with several decades of cognitive science research on decision-making in other domains. Numerous researchers have partitioned decisional processes into two competing systems, a “System 1” that is quick and reflexive and a “System 2” that is slow and deliberative (e.g., Gilbert, 1989; Sloman, 1996; Chaiken and Trope, 1999; Hammond et al., 1999; Evans, 2003; Kahneman, 2003). According to these researchers, however, System 1 is not solely the domain of emotion. Instead, intuition, heuristics, and experience-based biases are also considered vital parts of the rapid, reflexive System 1.

The singling out of emotion as the primary factor in early moral judgment is based on two lines of evidence. The first line depends on a theoretical commitment regarding the nature of utilitarian and deontological decision-making. Utilitarian reasoning judges the moral acceptability of an action based on its consequences, seeking to maximize the “greatest good for the greatest number.” In contrast, consequences are irrelevant to deontological concerns; instead the moral permissibility of an action depends on its adherence to purportedly universal moral rules (categorical imperatives). Greene et al. (2009) identified two particular deontological principles which, when violated, elicit a high proportion of deontological judgments. The first is whether the agent harms the victim in a manner that involves *personal force* (use of an agent’s muscles), and the second is whether the harmful outcome is intended or an unavoidable side effect of the action taken. An example of personal force would be pushing an individual onto the path of an oncoming trolley. Pushing a switch instead which diverts a trolley onto the individual’s path is impersonal. If diverting the trolley was done in order to deliberately kill the person, it is intentional harm. If instead, it was done to prevent the trolley from killing five people in its path, it is a forsee but unavoidable and unintended side effect. According to Greene et al. (2009), these two factors, when combined, yield the highest level of moral condemnation. As the authors put it “…personal force was found to interact with intention such that the personal force factor only affects moral judgments of intended harms, while the intention factor is enhanced in cases involving personal force.”

The second line of evidence consists of studies reporting activation of VMPC that is specific to moral judgments as compared to semantic judgments or other non-moral judgments (Greene et al., 2001; Moll et al., 2001; Heekeren et al., 2003). Importantly, when reasoning about moral dilemmas that pit deontological principles against utilitarian outcomes, individuals with damage to ventromedial frontal cortex (and hence impaired socio-emotional processing systems) are more likely to make utilitarian judgments than intact individuals or patients with damage to other areas of the brain. This outcome has been interpreted to mean that moral judgment becomes more deliberation-driven when affect-elicitation is impaired (Koenigs et al., 2007). Consistent with this interpretation, Greene et al. (2008) found that requiring participants to perform a concurrent digit-search task while making moral judgments selectively increased decision time for utilitarian judgments. They interpreted this to mean that it takes time and cognitive resources to be a utilitarian.

One implication of this dual-process analysis is that early moral judgments should be more affect-based while later ones should be more reasoning-based, a crucial prediction tested by Suter and Hertwig (2011) using dilemmas that pitted the welfare of a few against the welfare of many. Participants were required to make such moral decisions under unlimited-time or reduced-time conditions, and three types of deontological violations were employed: (a) high-conflict dilemmas, in which personal force was used to intentionally inflict harm, (b) low-conflict dilemmas, in which personal force was used to inflict harm that was an unintended side effect of the action taken, and (c) impersonal dilemmas, in which a harm was a side effect and no personal force was used. They found that restricting decision time reduced the number of utilitarian decisions for high-conflict vignettes, but not for low conflict or impersonal vignettes. These results are consistent with Greene’s dual-process model because truncating decision time deprived participants of the additional time needed to engage in deliberative (utilitarian) reasoning and/or to resolve decisional conflicts.

While consistent with Greene’s dual-process model, several factors hamper interpretation of Suter and Herwig’s (2011) results. First, the materials used were chosen because they differed in deontological criteria yet all yielded low inter-subject consensus in pilot work – each vignette elicited an equivalent number of “yes” and “no” judgments. These selection criteria ignored the most important factor in the fast vs. slow dual-process framework: decision time. If a vignette elicits a high degree of conflict between the two systems, then it should yield long decision times because it takes time to resolve conflicting system outputs. Because people differ in terms of how they choose to resolve the conflict, low inter-subject consensus results. Yet consensus and decision times reported by Greene et al., 2008, Supplementary Materials) did not necessarily show this pattern. For example, “Crying Baby,” which involves both personal force and intentional harm, yielded low inter-subject consensus (60% utilitarian judgments) and long mean decision time (5.7 s). But “Sophie’s Choice,” a dilemma that involves neither personal force nor intentional harm, also yielded low inter-subject consensus (62%) and long mean decision time (6.1 s). In contrast, “Donation,” a dilemma that also involves neither violation, yielded nearly identical low-inter-subject consensus (63%) but rapid mean decision time (4.8 s). For this reason, decision consensus may constitute an insufficient or misleading measure of hypothesized inter-system conflict.

Second, reading time was not controlled by Suter and Hertwig (2011) in a manner that ruled out the possibility that participants were devoting reading time to decision time. Participants were given a maximum of 35 s to read each vignette before the screen advanced to the decision question, yet no mention is made of vignette word count. For this reason, it is not clear whether decision time was held constant across vignettes.

These confounds were addressed in Experiment 1. Reading time was controlled in a scrolling format, and two methods were used to categorize vignettes as high or low decisional conflict. The first method categorized vignettes based on the *a priori* deontological factors proposed by Greene et al. (2009) and used by Suter and Hertwig (2011). The second was entirely empirically based. The dual-process model predicts that conflict between the two systems lengthens decision time because the conflict must be resolved. It also decreases inter-subject consensus because some people may resolve in favor of System 1 outputs while others may resolve in favor of System 2. Accordingly, *high decisional conflict vignettes* were defined as dilemmas that elicit long decision times and low inter-subject consensus. Conversely, *low decisional conflict vignettes* were defined as those that elicit fast decision times and high inter-subject decision consensus. *Moderate decisional conflict vignettes* were defined as those that elicit moderate decision times and moderate consensus. Decision time was manipulated, and the impact of restricting decision time on moral judgment was assessed. The model predicts that truncating decision time should preclude adequate deliberative processing, thereby shifting the balance of the judgments in favor of emotional outcomes. Because violations of deontological principles are predicted to elicit rapid negative affective responses, this means that early judgments should be more deontologically based than later ones. Moreover, vignettes that show long decision times and low inter-subject consensus should be exactly those that pit deontological outcomes against utilitarian ones.

A third confounding factor was the conflation of content with decisional processes, that is, the identification of deontology with System 1 and utilitarianism with System 2. Because no direct measure of the two systems was employed, this confound is a particularly serious one. In the materials employed by Suter and Hertwig (2011), all “yes” responses constituted utilitarian judgments and all “no” response constituted deontological ones. Matthews (2001) has argued that stressors, such as restricting decision time, may change the total quantity of decision-making resources available due to changes in (a) biological or neural functioning, (b) processing load (i.e., multiple tasks may overload the processing of information), or (c) strategic reallocation of processing resources (i.e., emotion-focused coping). An alternative interpretation of Suter and Herwig’s (2011) results, therefore, is that reducing decision time simply made people more likely to say “no” in order to cope with emotional or processing overload, not because they were resolving in favor of deontological concerns. To test this alternative explanation, decision queries in Experiment 2 were worded such that a “yes” or “no” response could be either a deontological decision or a utilitarian decision. If Greene’s dual-process model is correct, then the same results should obtain regardless of whether “yes” or “no” constitutes a utilitarian response.

Finally, the impact of emotion was specifically investigated in Experiment 3. A crucial aspect of the dual-process model proposed by Greene and colleagues is the role of emotion in moral judgment, not simply fast decisional processes. Emotion has been found to impact moral judgments when people are given ample time to make decisions, and the nature of the emotion elicited matters. The elicitation of disgust has been found to create harsher attitudes toward immoral behavior in general (Schnall et al., 2008), elicitation of joviality or mirth makes deontological violations seem more permissible (Valdesolo and DeSteno, 2006; Strohminger et al., 2011), and inducing feelings of “elevation” or benevolence makes such violations appear less permissible (Strohminger et al., 2011). Particularly relevant is the impact of stress on moral judgment. Youssef et al. (2012) used the Trier Social Stress Test (TSST) to induce stress (assessed by salivary cortisol levels). They found that activation of the stress response yielded a reduction in utilitarian responses that was specific to personal moral dilemmas that described deontological violations. The reduction in utilitarian judgments under conditions of time constraints (Suter and Hertwig, 2011) or concurrent task demands (Greene et al., 2008) may be simply due to the stress involved in conflict resolution.

To test this alternative explanation, participants in Experiment 3 read moral dilemmas and rated the pleasantness of esthetically pleasing photographs prior to rendering a decision. Reflecting on pleasant emotional stimuli is inconsistent with the hypothesized negative affect elicited by deontological violations. Performance of this group was compared to that of a control group who simply made decisions, and to a second control group who engaged in a cognitive distraction task based on the same stimuli. If deontological decisions are indeed strongly determined by rapid, negative affective responses, then inducing a pleasant emotional state during decision-making should decrease the frequency of deontological judgments in favor of utilitarian ones.

## Experiment 1

Greene’s dual-process model predicts that (a) early decisions should be emotion based while later decisions should be reason-based, and that (b) vignettes that describe deontological violations should elicit more emotion-based judgments. The purpose of Experiment 1 was to test these predictions. As in Suter and Hertwig (2011), the utilitarian structure of all dilemmas was the same: choose to sacrifice few to save many. They differed, however, in terms of decisional conflict profiles and in terms of deontological violations. Decision time was truncated for some subjects while others were given unlimited time to make their decisions. It was predicted that (a) vignettes whose decisional profiles are most strongly consistent with a conflict between the fast and slow systems should also be those that are most strongly impacted by a reduction in decision time, and (b) these same vignettes should also be those that describe deontological violations, thereby yielding strong emotional responses that are contrary to reasoned utilitarian aggregate benefit analyses.

## Methods and Materials

### Participants

Participants were 189 undergraduate students at the University of Illinois at Urbana-Champaign who participated in order to receive class credit. Sixty-four percent were female, and all ranged in age from 18 to 24. The median age for both females and males was 19.

### Materials and procedure

A total of 20 moral vignettes were used in the initial pool. Eighteen were selected from the battery of vignettes used in Greene et al., 2008, see the Supplementary Materials from that paper for decision times and consensus). These were “Crying Baby,” “Footbridge,” “Hard Times,” “Lawrence of Arabia,” “Sacrifice,” “Safari,” “Smother for Dollars,” “Sophie’s Choice,” Standard Fumes,” “Standard Trolley,” “Submarine,” “Transplant,” “Vaccine Policy,” “Vaccine Test,” “Vitamins,” “Modified LifeBoat,” “Modified Bomb,” and “Donation.” In all of these moral dilemmas, an action that benefits “the greater good” also has negative consequences for a minority of individuals. The percent utilitarian judgments for these vignettes ranged from 3 to 91%, and decision times ranged from 3 s to a little over 7 s. Two additional vignettes were used which were designed to elicit strong affect. “Cancer Toddler” was based on a dilemma used by Hauser et al. (2006). It describes a situation where five railway workers will die unless the participant redirects a runaway train toward their own terminally ill child. “Fumes with Friends” was a modification of the “Standard Fumes” vignette from Greene et al. (2008). Here, participants must choose between allowing toxic fumes to kill three hospital patients, or redirecting the fumes to another room where they will kill their best friend. This brought the total number of vignettes used to 20.

A custom E-Prime program was employed for the presentation of instructions and vignettes, and for the collection of participants’ responses. A session began with instructions in which participants were told they would be reading a series of stories and making decisions about them. They were then given a practice vignette. The order of the 20 experimental vignettes was randomized.

At the beginning of each trial, the computer displayed a white screen with gray letterboxes at the top and bottom of the screen. A black plus sign was located in the center of the screen to serve as a mask and to separate each trial. A counter indicating the trial number was located in the top letterbox, and instructions appeared in the bottom letterbox. Participants were instructed simply to press a key when they were ready to begin reading the first vignette.

Vignettes were presented as vertically scrolling text on the computer screen. The gray letterboxing remained at the top and bottom of the screen, creating a window in which the vignette text appeared. Only 2.5 lines of text were visible at a time. This controlled for reading speed by preventing participants from reading too far ahead in the vignette. After the entire vignette had been presented, participants were prompted to respond by the appearance of text on screen asking, “Is it appropriate to [take the action] in order to [produce the outcome]?” Participants indicated “yes” by pressing the 1 key on the number pad, and “no” by pressing the 3 key on the number pad.

Participants were randomly assigned to one of two conditions. In the *unlimited-time* condition, participants were allowed as much time as they wanted to respond. In the *restricted-time* condition, decision time was abbreviated to 200 ms for each word in the decision question, which resulted in an average of 4,400 ms. If no response was given with this time period, the gray letterboxes turned red, the words “Respond Immediately” appeared within both letterboxes, and two additional seconds were given for participants to respond. After these additional 2 s expired, or a response was recorded, a brief mask (a plus sign in the center of the screen) marked the end of the trial. Decision times were recorded at millisecond accuracy.

## Results and Discussion

Table 1 presents percent utilitarian decisions (decision consensus) and mean decision times for each vignette reported by Greene et al., 2008, Supplementary Materials) and obtained under conditions of unlimited decision time in Experiment 1, along with the vignettes’ deontological features. In the present study, percent utilitarian decisions ranged from 3 to 85%. There was nearly identical agreement between our subjects and those of Greene et al. (2008) on vignette decisions, *r* = 0.95, *t*(16) = 11.55, *p* < 0.001. Mean decision times ranged from 2,830 to 7,605 ms. The correlation between mean decision times in both studies for these vignettes was lower but still statistically significant, *r* = 0.47, *t*(16) = 2.15, *p* < 0.05.

**Table 1:** **Vignettes used in Experiments 1 and 2 ranked by % utilitarian responses**.

Vignette	Personal	Intention	% Util^a^	Dec. time (ms)^a^	% Util^b^	Dec. time (ms)^b^
Hard times	No	Intend	3	4,089	9	5,262
Transplant	Yes	Intend	5	2,830	12	3,047
Smother for dollars	Yes	Intend	8	3,766	7	4,242
Footbridge	Yes	Intend	15	3,107	21	4,288
Modified safari	No	Intend	28	6,455	22	5,442
Sacrifice	No	Intend	28	6,588	51	6,139
Fumes w/friends	No	Side effect	36	7,706	n/a	n/a
Vitamins	Yes	Side effect	38	5,602	35	6,352
Crying baby	Yes	Intend	40	6,366	60	5,651
Sophie’s choice	No	Intend	41	7,139	62	6,133
Cancer toddler	No	Side effect	52	7,366	n/a	n/a
Standard fumes	No	Side effect	62	4,935	76	4,417
Donation	No	Side effect	67	7,606	63	4,830
Modified lifeboat	Yes	Intend	67	3,742	71	4,526
Lawrence of Arabia	No	Intend	68	4,904	82	6,881
Vaccine test	No	Side effect	68	4,941	79	7,125
Vaccine policy	No	Side effect	75	5,694	85	6,164
Standard trolley	No	Side effect	80	4,176	82	4,335
Submarine	No	Intend	80	4,709	91	5,983
Modified bomb	Yes	Intend	85	4,690	90	7,073

*^a^Results from current study; ^b^Results from Greene et al. (2008)*.

### Vignette classifications

Given the moderate decision time correlation, we relied on decision times and consensus from our own subject population to classify vignettes into decisional conflict categories.

The vignettes were divided into five categories based on decisional conflict profiles as shown in Table 1. Starting from the top of Table 1, these were the category divisions. *Low Decisional Conflict-No vignettes* were those that elicited high “No” decision consensus and fast decision times; this category included “Hard Times,” “Transplant,” “Smother for Dollars,” and “Footbridge.” *Moderate Conflict-No vignettes* were those that elicited moderate “no” decisional consensus and moderately fast decision times; these were “Sacrifice,” “Fumes with Friends,” and “Vitamins.” *High Decisional Conflict vignettes* were defined as those that elicited low decision consensus and long decision times; this category included “Crying Baby,” “Sophie’s Choice,” and “Cancer Toddler.” *Moderate Conflict-Yes vignettes* were those that elicited moderate “Yes” decision consensus and faster decision times; these included “Standard Fumes,” “Modified Lifeboat,” “Lawrence of Arabia,” and “Vaccine Test.” *Low Decisional Conflict-Yes vignettes* were those that elicited fast decision times and high “yes” decision consensus; these were “Standard Trolley,” “Modified Bomb,” and “Submarine.” Three vignettes that could not be classified according to these criteria were excluded. They were “Modified Safari, “Vaccine Policy,” and “Donation.”

### Decision conflict analysis

**Figure 1:**
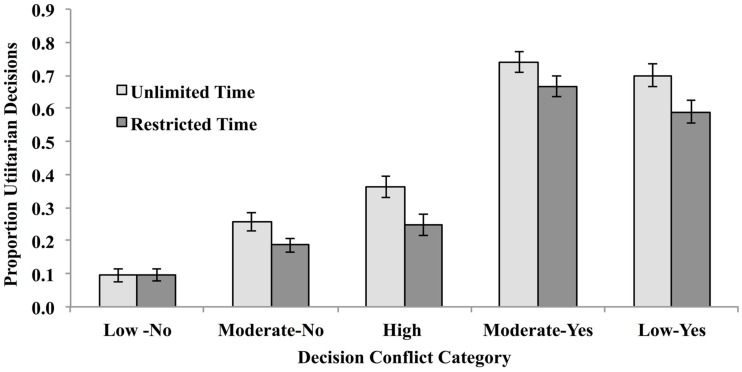
**Proportion utilitarian decisions made under unlimited- and reduced-decision time for high-conflict vignettes (long decision times and low inter-subject decision consensus), moderate conflict vignettes (moderate decision times and moderate inter-subject decision consensus), and low-conflict vignettes (fast decision times and high inter-subject decision consensus) in Experiment 1**.

Seven participants in the reduced-decision time condition were eliminated because they did not enter a decision for two or more vignettes. The remaining participants in this condition were able to reach a decision in 99.3% of the remaining trials. Participants therefore had ample time to reach a decision in the time allotted, and presumably only extended deliberation was interrupted. This strongly suggests that restricting decision time to 6 s did not impose an onerous cognitive load. The relevant means were substituted for the 0.7% (*n* = 10 trials) that had missing data. The resulting mean proportion utilitarian decisions are illustrated in Figure 1.

These mean proportions were analyzed via mixed ANOVA with decision time (Unlimited or Reduced) as a between subject variable and conflict (Low-No, Moderate-No, High, and Moderate-Yes, Low-Yes) as repeated measures. The main effects of decision time and conflict category were both significant, *F*(1,182) = 9.37, MSe = 0.16, *p* < 0.005, η^2^ = 0.05, and *F*(4, 728) = 277.42, MSe = 0.05, *p* < 0.00001, η^2^ = 0.60, respectively. These main effects were modified by a higher order interaction, *F*(4, 728) = 3.91, MSe = 0.05, *p* < 0.005, η^2^ = 0.02.

Planned comparisons indicated a pattern of results that differed from Suter and Hertwig (2011). As predicted by the dual-process model and as reported by Suter and Hertwig (2011), limiting decision time significantly reduced the number of utilitarian decisions in the High-Conflict condition, *t*(1,182) = 3.98, *p* < 0.0001 (two-tailed test), but had no impact on Moderate Conflict-No and Low Conflict-No categories, *t*’s(182) = 0.07 and 1.44, respectively, *p*’s > 0.05 (two-tailed test). But unlike Suter and Hertwig (2011), we found a significant reduction in “Yes” decisions in both the Moderate Conflict-Yes and Low Conflict-Yes categories, *t*’s(182) = 2.03 and 1.95, respectively, *p*’s < 0.05 (two-tailed tests). This suggests an across-the-board shift in strategy toward saying “no” under time pressure.

**Figure 2:**
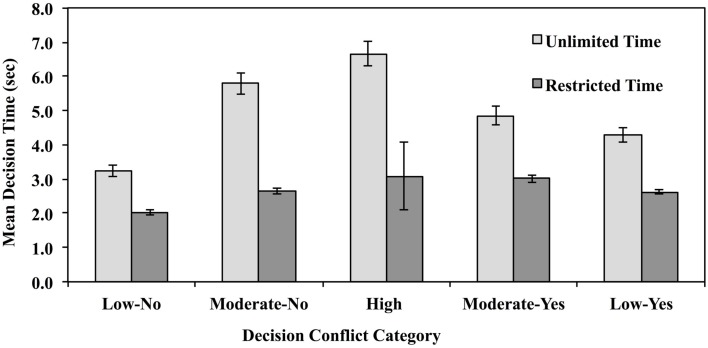
**Mean decision time for decisions made under unlimited- and reduced-decision time for high-conflict vignettes (long decision times and low decision consensus), moderate conflict vignettes (moderate decision times and moderate inter-subject decision consensus), and low-conflict vignettes (fast decision times and high inter-subject decision consensus) in Experiment 1**.

Decision times were analyzed in order to ensure that time restriction did in fact result in shorter decision times relative to the unlimited-time condition. If it had no effect, this would invalidate the intent of the manipulation, which was to reduce opportunity for deliberation and/or conflict resolution. Decision times were trimmed to eliminate times that equaled or exceeded 3 SD from the mean within the appropriate Conflict × Decision Time cell. This resulted in the elimination of 1.22% of the data. Remaining mean decision times were calculated for each subject within each decisional conflict category. These are presented in Figure 2.

Mean decision times were analyzed via mixed ANOVA with condition (Unlimited or Reduced Time) as a between subject variable and conflict (Low Conflict-Yes, Moderate Conflict-Yes, High Conflict, Moderate Conflict-No, and Low Conflict-No) as repeated measures. The main effect of decision time was significant, as was the main effect of conflict, *F*(1,182) = 107.67, MSe = 12,578, *p* < 0.0001, η^2^ = 0.37, and *F*(2,728) = 68.76, MSe = 2,188, *p* < 0.0001, η^2^ = 0.27. The interaction of these variables was also significant, *F*(2, 728) = 22.96, MSe = 2,188, η^2^ = 0.11, *p* < 0.00001.

Planned comparisons indicated that people made quicker decision times in the reduced-time condition than in the unlimited-time condition regardless of conflict category, *t*’s(182) = 7.83, 8.57, 9.75, 7.07, and 7.60 for the Low Conflict-No, Moderate Conflict-No, High Conflict, and Moderate Conflict Conflict-Yes and Low Conflict-Yes categories, respectively, *p*’s < 0.0001. Hence, people did indeed take longer to reach decisions in each conflict condition – not just the High-Conflict category – when allowed to take as much time as they wanted.

As is apparent from Figure 2, the pattern of decision times is not entirely consistent with dual-process model predictions. If utilitarian judgments require more deliberative thought, then “yes” decisions should take longer than “no” decisions, and high decisional conflict judgments should take the longest time of all conflict categories. Looking first at the unlimited-time condition, planned comparisons showed that Low Conflict-Yes decisions did in fact take longer than Low Conflict-No decisions, *t*(91) = 7.45, *p* < 0.0001, but the opposite was true for Moderate Conflict decisions, *t*(91) = −5.61, *p* < 0.0001. Moderate Conflict-No decisions took as long as High-Conflict decisions, *t* < 1. The same pattern is apparent in the data published by Greene et al. (2008). Mean decision time for vignettes that elicited the fewest utilitarian decisions (“Hard Times,” “Transplant,” and “Smother”) was 4.18 s; for vignettes that elicited the greatest number (“Modified Bomb,” “Submarine,” “Standard Trolley,” and “Lawrence of Arabia”), mean decision time was 6.07 s. But for personal dilemmas that elicited moderate decisional conflict, the opposite was true; “no” responses required an average of 5.36 s (“Footbridge,” “Modified Safari,” and “Vitamins”) “yes” responses required an average of 4.47 s (“Standard Fumes” and “Modified Lifeboat”).

Turning now to the reduced-time condition, Low Conflict-Yes decisions required more time (*M* = 2.96) than Low Conflict-No decisions (*M* = 2.10) *t*(91) = 10.71, *p* < 0.0001. Moderate-Yes decisions (*M* = 2.85) took no longer than Moderate-No decisions (*M* = 2.88), *t* < 1. High-Conflict decisions (*M* = 6.78) took longer than any other decision [High vs. Low-Yes *t*(91) = 4.36, *p* < 0.0001, High vs. Low-No *t*(91) = 12.89, *p* < 0.0001, High vs. Moderate-Yes *t*(91) = 5.01, *p* < 0.0001, High vs. Moderate-No *t*(91) = 5.08, *p* < 0.0001]. Thus, when decision time was truncated – and deliberation thereby cut short – utilitarian decisions tended to take longer than deontological decisions, and resolving decisional conflicts took longest of all.

#### Summary of decisional conflict analyses

Reducing decision time lowered the proportion of “yes” decisions for vignettes that usually elicit “yes” responses. It also lowered the proportion of “yes” decisions for vignettes that normally elicit nearly equivalent numbers of “yes” and “no” responses and hence implicate high decisional conflict. Vignettes that typically elicit “no” responses were unaffected by reductions in decision time. This pattern of results is predicted by the dual-process model in that saying “yes” is described as a utilitarian judgment that requires deliberative reasoning. But decision time for “no” responses took longer than “yes” responses when the vignette profiles indicated moderate conflict. This is inconsistent with dual-process predictions.

### Deontological principles

The results of the decision conflict analysis would be consistent with the dual-process model proposed by Greene (2007) if two conditions were true. First, deontological decisions must be rapid ones, that is, they must be the domain of System 1. Second, a shift in strategy from saying “yes” to saying “no” must reflect a strategic change from taking time to resolve deontological and utilitarian concerns to relying solely on fast deontological outputs.

A deontological analysis was conducted in order to test these predictions. According to the dual-process model, vignettes that described deontological violations (using personal force to inflict intentional harm) should induce long decision times and low decision consensus. This is because the fast, deontological system outputs a “no” response while the slower, utilitarian system outputs a “yes” response, and resolving the conflict takes time and cognitive resources. In contrast, those that describe no such violations yield no such conflict, hence there is little need for time- and resource-demanding conflict resolution. This was the reasoning used by Suter and Hertwig (2011) when classifying vignettes as “high” or “low” conflict.

As is apparent from the data presented in Table 1, participants’ decision times and decision consensus did NOT reflect differences in deontological violations. “Crying Baby,” “Cancer Toddler,” and “Sophie’s Choice” all showed low inter-subject consensus and long decision times, yet only the first of these (“Crying Baby”) involves personal force and intentional harm. Of the four vignettes that elicited the fastest “no” decisions, one involves neither type of violation (“Hard Times”); conversely, of the four that elicited the fastest “yes” decisions, one described both types of violations (“Modified Bomb”), one involved no personal force (“Submarine”), and two described neither type of violation (“Vaccine Policy” and “Standard Trolley”). This means that the deontological criteria of personal force and intention were not entirely predictive of decision time or inter-subject decision consensus. Other unidentified factors apparently contribute to making decisions slow or fast, difficult or easy.

To better assess the relationship between deontic violations and decision profiles, two regression analyses were conducted. Vignettes were assigned a score of 2 if both principles were violated (*n* = 6 vignettes), a score of 1 if only one principle was violated (*n* = 6 vignettes), and a score of 0 if none were violated (*n* = 6 vignettes). (Impersonal vignettes “Donation,” and “Vaccine Policy” were excluded.) The proportion utilitarian decisions in the unlimited-time condition was then regressed onto deontological score. The regression was not significant, *F* < 1, adjusted *R*^2^ = −0.02. Mean decision times were also regressed onto deontological score, and this regression was also not significant, *F*(1,16) = 3.31, *p* = 0.09, adjusted *R*^2^ = 0.12. Thus, factors other than personal force and intention contribute to decision consensus and decision times.

**Figure 3:**
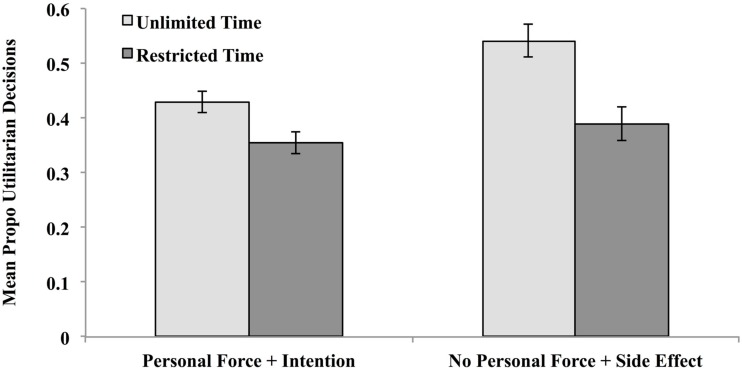
**Proportion utilitarian (“Yes”) decisions made as a function of decision time (unlimited or reduced) for vignettes that involve the use of personal force to intentionally produce harm and for vignettes that do no involve the use of personal force but yield harm as an unintended side effect in Experiment 1**.

To test this more precisely, vignettes were re-classified into two categories. The first included five vignettes that described the use of personal force to inflict intended harm (PF-I): “Footbridge,” “Lifeboat,” “Smother for Dollars,” “Modified Bomb,” and “Crying Baby.” (“Transplant” was excluded as surgery did not seem to us to involve personal force to the same degree as the others, even though it is “up close and personal.”) Using Suter and Hertwig’s classification scheme, these would constitute “High-Conflict Personal” vignettes. The second category included five vignettes that clearly describe actions in which the harm involved no personal force and was a side effect rather than an intended outcome (NPF-SE): “Standard Fumes,” “Fumes with Friends,” “Cancer Toddler,” “Standard Trolley,” and “Sophie’s Choice.” (“Donation” and “Vaccine Policy” were excluded because they were not “personal,” “Hard Times” was excluded because no death was involved, unlike all the others, and “Vaccine Test” was excluded because it involves the use of a syringe.) Using the classification scheme of Hertwig and Suter, these would constitute “Low-Conflict Personal” vignettes. The proportion utilitarian (“yes”) decisions were calculated for each subject for these two sets of vignettes, and they are depicted in Figure 3.

The dual-process model and the results of Suter and Hertwig (2011) predict that these two categories should yield the most divergence in decision consensus. Four planned comparisons were conducted to test these predictions. As predicted, when participants were allowed to take as much decision time as they wanted, more “yes”/utilitarian decisions obtained when no deontological violations were described, mean NPF-SE = 0.54 vs. mean PF-I = 0.43, *t*(91) = 3.64, *p* < 0.0001. When decision time was reduced, however, no difference obtained, mean PF-I = 0.36 vs. mean NPF-SE = 0.39, *t*(91) = 1.21, *p* = 23. This was because time truncation had less impact on vignettes that involved violations (0.43–0.46) than on vignettes that did not involve violations (0.54–0.39), *t*(182) = 1.88, *p* = 0.03 (one-tailed test).

**Figure 4:**
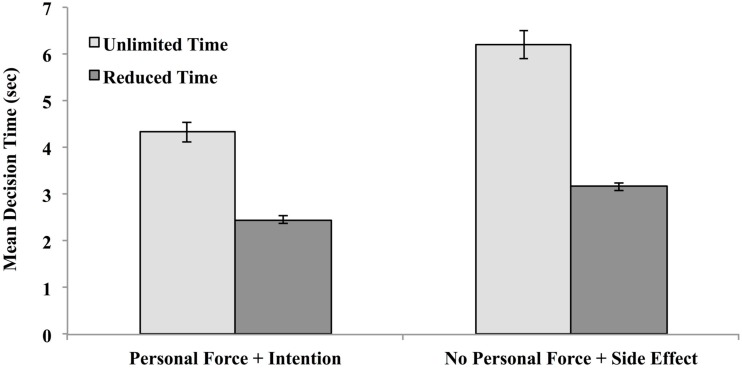
**Mean decision time for utilitarian (“Yes”) decisions as a function of decision condition (unlimited or reduced-decision time) for vignettes that involve the use of personal force to intentionally produce harm and for vignettes that do no involve the use of personal force but yield harm as an unintended side effect in Experiment 1**.

The same analysis was done on mean decision times, which are depicted in Figure 4.

Contrary to dual-process predictions, when given as much decision time as they wanted, participants took significantly less time to render decisions for vignettes involving personal force and intentional harm (*M* = 4.33 s) than for vignettes that did not involve such violations (*M* = 6.19 s), *t*(91) = 7.29, *p* < 0.001. When decision time was truncated, they still took less time when deontological violations were involved (*M* = 2.45 s) than when they were not involved (*M* = 3.17), *t*(91) = 9.23, *p* < 0.0001. Yet these deontological violations should have put these vignettes in greatest decisional conflict with their utilitarian properties.

#### Summary of deontological analyses

People quickly reject courses of action that require intentionally harming someone through the use of personal force. When neither principle is violated, they require more time to make a moral judgment. As a result, truncating decision time had a stronger impact on vignettes that, according to Greene’s (2007) model, require more deliberation and/or conflict resolution between conflicting deontological and utilitarian outputs. Decisional conflict, however, could not be predicted solely on the basis of personal force and intentional harm. Other vignette features appear to contribute to decisional conflict as measured by decision time and consensus. Moreover, conflicts between deontological and utilitarian outcomes yielded faster rather than slower decision times. This pattern of results is consistent with two alternative decision-making strategies: (a) saying “no” under time pressure or (b) using deontological principles as heuristics.

## Experiment 2

The results of Experiment 1 are inconsistent with Greene’s (2007) claim that time is needed to overcome a prepotent emotional response, but they are also consistent with two other explanations. The first is that under time pressure, people are more likely to say “no” to difficult dilemmas than they are to say “yes.” In the studies cited in our introduction and in Experiment 1 above, saying “no” was not only equivalent to doing nothing, it also constituted a deontological response. The apparent rise in deontological judgments may have in fact have reflected nothing more than people saying “with so little time to decide, I choose to do nothing.” Hence, it was imperative to decouple “no” and “deontological judgment” to see if people really were making more deontological judgments; Experiment 2 addressed that confound, and ruled out that alternative explanation. The second is that deontological principles constitute heuristic rules, allowing rapid decisions to be made, and have little to do with emotion.

Experiment 2 addressed the first of these alternate explanations – that decision time pressure yields a strategy shift toward simply saying “no.” Participants again made moral judgments under both unlimited-time and restricted-time conditions. They were required to make a rapid response (within 6 s), and then were given the opportunity to deliberate as long as they wanted. Half of the participants were queried in a way that “no” responses were consistent with deontological principles while “yes” responses were consistent with utilitarian principles. The remaining participants were queried in such a way that the reverse was true.

## Methods and Materials

### Participants

Sixty-five students at the University of Illinois served as participants in the study. They were recruited via advertisement on the Psychology Department website, and were paid $5 for their participation. Sixty-two percent were female, and ages ranged from 18 to 21.

### Materials

The same vignettes were used as in Experiment 1. The trial driver consisted of a Qualtrics survey run on dedicated iMac computers. The instruction screen stated the following: “Before each story, you will see a prompt that says “READY?” and an arrow. When you are ready to begin, click on the arrow, then place your cursor on the X on the next screen. The story will begin 2 s after the X appears. You will have 6 s to make your first decision about each story. IT IS VERY IMPORTANT THAT YOU ENTER A DECISION WITHIN THIS TIME FRAME. A timer will be displayed in the upper left corner of the screen so you know how much time you have left. You will then be given as much time as you need to enter a second decision. The two decisions may end up being the same, or you may decide you would like to change your mind.” The first decision prompt displayed the label “First Decision,” a count-down timer, the question, and the choices “Yes” and “No.” Participants entered their decision by clicking on one of the choices. If a decision was not made within the allotted time, the screen advanced to the next screen. This screen showed the label “Second Decision,” the question and the same choices.

The questions in the “Take Action” condition all took the following form: “Is it appropriate to <take the described action> under the circumstances.” A “yes” response is consistent with a utilitarian judgment, and a “no” response is consistent with a deontological judgment. The questions in the “Refuse to Take Action” condition all took the following form: “Is it appropriate to REFUSE to <take described action> under the circumstances?” Here, a “no” response is consistent with a utilitarian judgment, and a “yes” response is consistent with a deontological judgment.

## Results and Discussion

### Decisional conflict analyses

**Figure 5:**
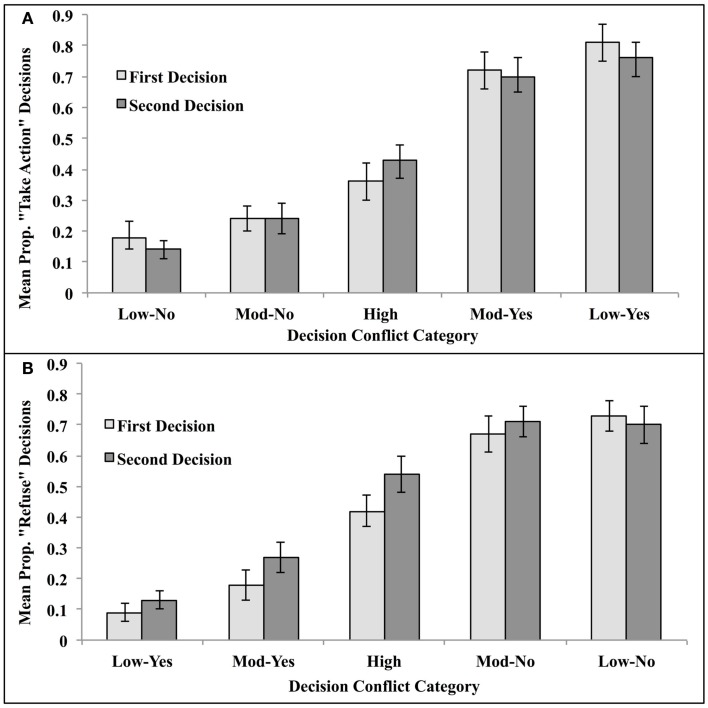
**Proportion utilitarian decisions made under unlimited- and reduced-decision time for high decisional conflict vignettes, moderate conflict vignettes, and low-conflict vignettes when the moral judgment was queried as (A) “Is it appropriate to <take the described action>?” or (B) “Is it appropriate to refuse to <take the described action>?” in Experiment 2**. A “yes” response constituted a utilitarian decision for the former query, while a “no” response constituted a utilitarian decision for the latter.

Vignettes were classified as in Experiment 1, and mean proportion utilitarian decisions for each category were calculated. They are depicted in Figure 5. Decision times were analyzed as in Experiment 1. They were trimmed to eliminate times that equaled or exceeded 3 SD from the mean within the appropriate Conflict × Decision Time cell. This resulted in the elimination of 1.85% of the data. Remaining mean decision times were calculated for each subject within each conflict condition. These are presented in Figure 5.

These means were analyzed via mixed ANOVA with question type (Take Action or Refuse to Take Action) as a between subject variable; decisional conflict category (Low-No, Moderate-No, High, and Moderate-Yes, Low-Yes), and decision trial (First and Second) served as repeated measures. The main effect of conflict category was significant, *F*(4, 252) = 98.30, MSe = 0.10, *p* < 0.0001, η^2^ = 0.61, and, as predicted, it was modified by a significant interaction with trial, *F*(4,252) = 3.65, MSe = 0.02, *p* < 0.006, η^2^ = 0.06. Importantly, this interaction was *not* modified by question type, *F* < 1. This means that it did not matter whether “yes” or “no” constituted a utilitarian judgment.

Planned comparisons indicated that, as predicted by the dual-process model, limiting decision time significantly reduced the number of utilitarian decisions in the High-Conflict condition, *t*(1,64) = 2.72, *p* < 0.01 (two-tailed test). No other comparison was significant. Given our within-subject design, this means that, when given the opportunity to deliberate further, participants changed their deontological responses to utilitarian responses ONLY for high decisional conflict dilemmas.

The interaction of question type and trial was significant, *F*(1, 63) = 5.11, MSe = 0.03, *p* < 0.03, η^2^ = 0.08. This interaction was due to the “refuse to take” query form imposing a decisional load separate from other factors. When asked whether to refuse to take the stated action, participants increased their utilitarian decisions from the first trial (*M* = 0.42) to the second (*M* = 0.47), simple effects *F*(1, 32) = 5.19, MSe = 0.01, *p* < 0.05. No increase obtained when they were asked whether to take the action, *M* first = 0.46, *M* second = 0.45, simple effects *F* < 1. This interaction was not modified by conflict category, *F* < 1, indicating that the effect held regardless of decisional conflict profiles.

**Figure 6:**
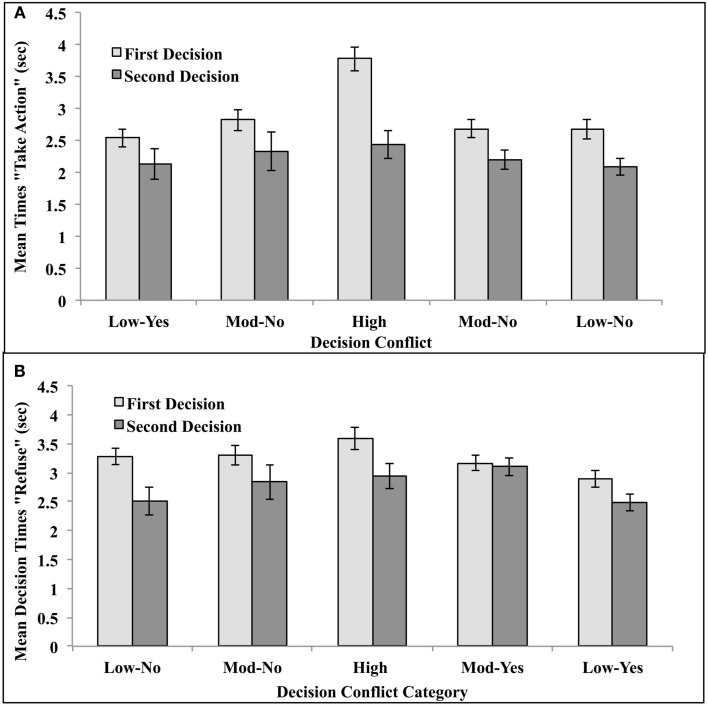
**Mean decision time for decisions made under unlimited- and reduced-decision time for high-conflict vignettes, moderate conflict vignettes, and low-conflict vignettes in Experiment 2**. First decisions were made within a time constraint of 6 s. Second decisions were made without time constraints. Moral judgments were queried as **(A)** “Is it appropriate to <take the described action>?” or **(B)** “Is it appropriate to refuse to <take the described action>?” A “yes” response constituted a utilitarian decision for the former query, while a “no” response constituted a utilitarian decision for the latter.

Decision times were trimmed as in Experiment 2. Mean times are presented in Figure 6.

Mean decision times were analyzed via mixed ANOVA with question type (Take Action or Refuse to Take Action) as a between subject variable, and trial (First or Second Decision) and conflict category (Low Conflict-Yes, Moderate Conflict-Yes, High Conflict, Moderate Conflict-No, and Low Conflict-No) as repeated measures. The main effects of conflict category and trial were both significant, as was their interaction *F*(4, 252) = 10.07, MSe = 0.85, *p* < 0.0001, η^2^ = 0.15, *F*(1, 63) = 16.61, MSe = 3.13, *p* < 0.001, η^2^ = 0.21, *F*(4, 252) = 4.13, MSe = 0.63, *p* < 0.003, η^2^ = 0.06, respectively. The main effect of question type was NOT significant, *F* < 1. This means that the results of Experiment 1 were not due to a conflation of “yes” with utilitarian decisions and “no” with deontological decisions.

Planned comparisons indicated that when decision time was restricted on the first trial, people took longer to make decisions about High-Conflict vignettes than for any other type of vignette: High Conflict vs. Moderate Conflict-Yes vignettes, *t*(64) = 6.23, *p* < 0.001, Moderate Conflict-No vignettes, *t*(64) = 5.52, *p* < 0.001, Low Conflict-Yes vignettes, *t*(64) = 5.14, *p* < 0.0001, and Low Conflict-No vignettes, *t*(64) = 9.57, *p* < 0.001. When given the opportunity to reflect and deliberate on the second trial, they spent more time thinking about High-Conflict vignettes than they did Low-Conflict vignettes regardless of final decision: Conflict-Yes vignettes, *t*(64) = 2.39, *p* < 0.05; for Low Conflict-No vignettes, *t*(64) = 1.98, *p* < 0.05. (There were no differences between the additional time taken for High Conflict and Moderate Conflict-Yes or Moderate Conflict-No vignettes, *t*’s < 1.)

#### Summary of decisional conflict analyses

As in Experiment 1, people rendered fewer utilitarian judgments for dilemmas that induce high decisional conflict when decision time was truncated. When given more time, they were more likely to change their deontological judgments into utilitarian ones. They also required more time to make utilitarian judgments when decision time was truncated and when they were given unlimited time to decide. These results held regardless of whether a “yes” or “no” decision constituted a utilitarian judgment.

### Deontological analyses

As in Experiment 1, vignettes were re-classified using personal force and intention as discriminating features. A mixed ANOVA was conducted on proportion utilitarian decisions using question type (Take Action or Refuse to Take Action) as a between subject variable, and decision time (Restricted and Unlimited) and deontic classification (Personal Force + Intention and No Personal Force + Side Effect) as repeated measures. The analysis returned a significant interaction of trial and deontic classification, *F*(1,63) = 8.18, MSe = 0.01, *p* < 0.01, η^2^ = 0.12. When people were given the opportunity to further deliberate about dilemmas that did not violate deontological principles, they reliably increased the number of utilitarian decisions they made from 0.48 to 0.54, *t*(64) = 2.41, *p* < 0.025. Having a second chance to think about their decisions had no reliable effect, however, when the dilemmas violated deontological principles, 0.51 vs. 0.49, *t*(64) = 1.35, *p* = 0.18. This result would seem to suggest that deontological judgments are either driven solely by application of heuristic rules or influenced strongly by prepotent emotional responses, while utilitarian decisions constitute outcomes of deliberative conflict resolution processes.

The interaction of trial and question type was significant, *F*(1,63) = 8.12, MSe = 0.02, *p* < 0.01, η^2^ = 0.11. When asked whether it was appropriate to refuse to take the stated action, the proportion of utilitarian decisions increased with additional decision time from 0.47 to 0.53, *t*(32) = 2.53, *p* < 0.025. When asked whether it was appropriate to take the stated action, allowing additional decision time had no reliable effect [0.51 for the first decision and 0.49 for the second, *t*(31) = 1.46, *p* = 0.15].

**Figure 7:**
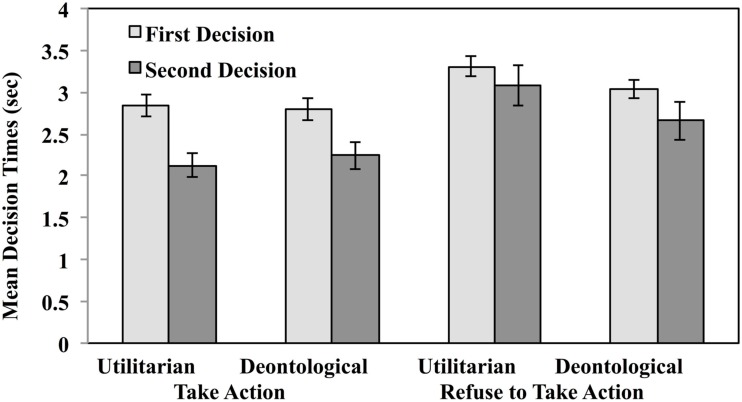
**Mean decision times for utilitarian and deontological judgments as a function of question type in Experiment 2**. First decisions were made within a time constraint of 6 s. Second decisions were made without time constraints.

Turning now to decision times, Figure 7 illustrates mean decision times for first and second decisions as a function of question type.

An ANOVA based on the same factors returned three significant effects: the main effect of deontic classification *F*(1,63) = 5.92, MSe = 0.38, *p* < 0.02, η^2^ = 0.09, the main effect of trial, *F*(1,63) = 28.20, MSe = 1.19, *p* < 0.0001, η^2^ = 0.31, and the main effect of question type, *F*(1,63) = 4.87, MSe = 2.12, *p* < 0.03, η^2^ = 0.07. These results meant that people took longer to make decisions when the question asked whether to refuse to take action (*M* = 3.02 s) than whether to take action (*M* = 2.62 s), they took more time to make their first decision (*M* = 3.18 s) than to make their second decision (*M* = 2.46), and to make judgments when no deontological principles were violated (*M* = 2.91) than when they were violated (*M* = 2.72). This last effect, again, is contrary to dual-process predictions.

#### Summary of deontological analyses

The overall pattern of results indicate that (a) it is easier to think about whether to take action than whether to refuse to take action, and (b) dilemmas that violate deontological principles yield faster decisions than dilemmas that do not. They also show that reducing decision time has greater impact on dilemmas that comprise a conflict between fast and slow decisional processes such that fewer utilitarian judgments obtain.

### Experiment 3

The results of Experiments 1 and 2 clearly show dilemmas that induce decisional conflict between deontological and utilitarian principles impact decision time as well as decision consensus. Limiting decision time shifts the decisional balance in favor of deontological judgments, but these results do not necessarily implicate the role of emotion in the deontological reasoning process. The fast System 1 identified by Kahneman (2003) and others also includes rule-based heuristic decision-making and intuition. The fast deontological decisions our participants displayed may have been due to the invocation of heuristic deontological rules, such as never using a person as an object (as is done when using personal force) or never intentionally causing another being harm. This is supported by the fact that deontological violations were associated with faster decision times. As mentioned previously, Youssef et al. (2012) found that activation of the stress response yielded a reduction in utilitarian responses that was specific to personal moral dilemmas that described deontological violations. The reduction in utilitarian judgments under conditions of time constraints (Suter and Hertwig, 2011) or concurrent task demands (Greene et al., 2008) may be simply due to the stress involved in conflict resolution.

The purpose of Experiment 3 was to investigate the separate impact of emotion and cognition on rapid decisional outputs. Participants made decisions under time constraints for a subset of moral dilemmas that normally elicit distress. Midway through the allotted decision-making time, one group of participants rated photographs selected to elicit pleasant emotional states while a second performed a cognitive task on the same photographs. They then delivered their judgments. We predicted that shifting attention to pleasant emotional stimuli would ameliorate emotional stress thereby freeing up vital decision-making resources and hence decrease the number of deontological decisions in favor of utilitarian ones.

## Methods and Materials

### Participants

One-hundred thirty-five undergraduate students at the University of Illinois at Urbana-Champaign served as participants in the study. Fifty-eight percent were female, and ages ranged from 18 to 21. An equal number (*n* = 45) participated in the control, emotion rating, and cognitive task groups. Participants were paid $5 for their participation.

### Materials

Vignettes from the previous studies were selected for use based on the following criteria: High and moderate decisional conflict as evidenced by decision times and decision consensus, violations of personal force and intentional harm (PF-I), and no personal force and harm as a side effect (NPF-SE). These criteria yielded the following vignettes which served as materials: Crying Baby (High Decisional Conflict, PF-I), Sophie’s Choice (High Decisional Conflict, NPF-SE), Vitamins (Moderate Decisional Conflict-No, PF-I), Fumes with Friends (Moderate Decisional Conflict-No, NPF-SE), Lifeboat (Moderate Decisional Conflict-Yes, PF-I), and Vaccine Test (Moderate Decisional Conflict-Yes, NPF-SE). In addition, “Modified Safari” served as an initial practice problem, and “Cancer Toddler” served as the last (unscored) problem.

Distractor materials consisted of photographs of pleasant houses in attractive landscaping. The houses differed in terms of number of windows, ranging from 6 to 12.

### Procedure

The trial driver was a Qualtrics survey run on dedicated iMac computers. A control group simply read each vignette and entered a decision within a restricted-time period. The instruction screen contained the following information: “You will read eight stories. Before each story, you will see a prompt that says “READY?” and an arrow. When you are ready to begin, click on the arrow, then place your cursor on the X on the next screen. The story will begin 2 s after the X appears. It will scroll up through the window at a comfortable reading rate.”

“When the story ends, you will have 4 s to think about it. A timer will be displayed in the upper left corner of the screen so you know how much time you have left. Lastly, you will be have 20 s to enter a decision about the story you read. The timer will also be displayed.” Safari and Lawrence of Arabia served as practice dilemmas, and were always the first second dilemmas displayed. The remaining six dilemmas were presented in random order. The questions asked were the same as the positive format questions in Experiment 2. Participants entered their decisions by clicking on either “yes” or “no” radio buttons.

The emotion group was given the following instructions: “You will read eight stories. Before each story, you will see a prompt that says “READY?” and an arrow. When you are ready to begin, click on the arrow, then place your cursor on the X on the next screen. The story will begin 2 s after the X appears. It will scroll up through the window at a comfortable reading rate.”

“When the story ends, you will have 4 s to think about it. A timer will be displayed in the upper left corner of the screen so you know how much time you have left. Then you will see a picture of a house. You will be asked to rate the house in terms of how pleasing you find it to be. You will have 10 s to give your rating, and a timer will be displayed. Lastly, you will have 10 s to enter a decision about the story you read and a timer will be displayed.” The house rating trials consisted of a color photograph of a house with a rating scale beneath it. The scale consisted of radio buttons that were labeled Very Unpleasant, Unpleasant, Somewhat Unpleasant, Neutral, Somewhat Pleasant, Pleasant, and Very Pleasant. Participants entered their decisions by clicking on a radio button that reflected their judgment.

The cognitive group was given the following instructions: “You will read eight stories. Before each story, you will see a prompt that says “READY?” and an arrow. When you are ready to begin, click on the arrow, then place your cursor on the X on the next screen. The story will begin 2 s after the X appears. It will scroll up through the window at a comfortable reading rate.”

“When the story ends, you will have 4 s to think about it. A timer will be displayed in the upper left corner of the screen so you know how much time you have left. Then you will see a picture of a house. You will be asked to count the number of windows in the house and multiply the total by three. You will have 10 s to record your answer and a timer will be displayed. Count WHOLE windows, not window panes. Count only windows that can be opened as a unit.” This was followed by three photographs of multi-paned windows captioned “This is counts as <> windows.” This was done to ensure that no confusion would result in participants counting window panes rather than windows. The instructions then continued. “Lastly, you will be have 10 s to enter a decision about the story you read and a timer will be displayed.” Participants entered their window answers by selecting from among four multiple choice options.

## Results and Discussion

### Decisions

**Figure 8:**
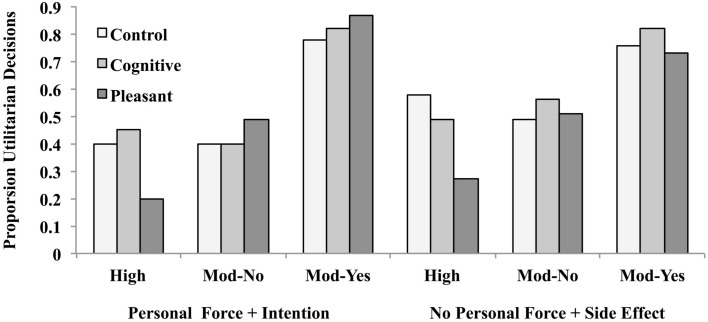
**Proportion utilitarian judgments following an emotion rating task involving pleasant stimuli, a cognitive task requiring mental arithmetic, and a control group that involved neither in Experiment 3**. Dilemmas either involved the use of personal force to inflict intentional harm, or no personal force where harm was an unintended side effect, and were pre-classified as involving high or moderate decisional conflict.

The proportion utilitarian judgments for each condition is depicted in Figure 8.

The proportion observed for the control condition was used as the expected probability of a utilitarian response. If rapid, negative affective responses are primarily responsible for deontological judgments, then disruption of negative emotional processing through exposure to pleasant stimuli should reduce their number relative to utilitarian judgments. This should be particularly true for high decisional conflict dilemmas that pit deontic violations against utilitarian concerns. In fact, the opposite obtained: For the high-conflict dilemma that involved personal force and intentional harm (Crying Baby), 40% of the control group (18 out of 45) gave utilitarian judgments. In the emotion rating condition, 20% (9 out of 45) gave a utilitarian decision, which constituted a significant *reduction* in utilitarian judgments, *Z*= −2.59, *p* < 0.01. In the cognitive condition, 45% (20 out of 51) gave a utilitarian judgment, which did not differ from the baseline control, *Z* = +0.45, *p* = 0.64. This pattern suggests that rapid judgments for this type of dilemma are primarily driven by System 1, non-deliberative processing, and that reducing or disrupting negative affect processing make people *more likely* to render a deontological judgment.

The same pattern of results obtained for the dilemma that did not involve personal force or intentional harm (Sophie’s Choice). Here, 58% (26 out of 45) gave a utilitarian response in the control condition. In the emotion condition, only 27% (12 out of 45) gave a utilitarian judgment, which also constituted a significant reduction in such judgments *Z* = −4.11, *p* < 0.0001. Among participants who engaged in the cognitive task, 49% (22 out of 45) gave a utilitarian judgment, which did not differ from the expected proportion *Z* = −1.09, *p* = 0.27. Thus, inducing a pleasant emotional state again shifted the decisional balance in favor of deontological judgments.

Turning now to Moderate Conflict-Yes dilemmas, we found the following. For the dilemma that violated the two deontological principles (Lifeboat), 76% of the control group (34 out of 45) gave utilitarian judgments, and neither the emotion (73%) nor the cognitive manipulation (82%) yielded significant changes from baseline, both *Z*’s = −0.24 and +0.80, *p*’s = 0.42 and 0.78, respectively. The same was true for the dilemma that did not involve deontological violations (Vaccine Test); here 78% of the control group (33 out of 45) gave utilitarian judgments. The percentages for the emotion and cognitive task groups were 82% (37 out of 45) and 84% (38 out of 45), *Z*’s = +1.23 and +1.56, *p*’s = 0.21 and 0.11, respectively.

Turning lastly to Moderate Conflict-No dilemmas, we found the following. For the dilemma that violated the two deontological principles (Vitamins), 49% of the control group (22 out of 45) gave utilitarian judgments, and neither the emotion (51%, 23 out of 45) nor the cognitive manipulation (56%, 25 out of 45) yielded significant changes from baseline, Z’s = +0.13 and +0.73, *p*’s = 0.89 and 0.46, respectively. For the dilemma that did not involve deontological violations (Fumes with Friends), 40% of the control group (18 out of 45) gave utilitarian judgments. Here, again, neither the emotion nor the cognitive task impact judgment values (emotion 49%, *n* = 22 out of 45, *Z* = +1.07, *p* = 0.28; cognitive 40%, *n* = 18 out of 45, *Z* = 0, *p* = 1).

While the results of the decision analysis indicates the emotion distractor task impacted high-conflict judgments, an alternative explanation is that the groups differed in terms of the amount of time they spent making decisions after they completed their tasks. To rule out this possible explanation, decision times were analyzed, and specific comparisons were made.

Decision times were analyzed via mixed ANOVA using distractor condition (Emotion, Cognitive, or Control) as a between subject variable, and vignette decisional conflict (High, Moderate-Yes, and Moderate-No) and deontology (Personal Force + Intention and No Personal Force + Side Effect) as repeated measures. The main effects of distractor condition, decisional conflict, and deontology were all significant, *F*(2, 132) = 81.49, MSe = 11.86, *p* < 0.0001, η^2^ = 0.56; *F*(2, 264) = 13.28 MSe = 7.08, *p* < 0.0001, η^2^ = 0.09; *F*(1, 132) = 7.62, *p* < 0.0001, η^2^ = 0.17. These main effects were modified by higher order interactions, Distractor Condition × Conflict *F*(4, 264) = 4.70, *p* < 0.001, η^2^ = 0.07; Distractor Condition × Deontology *F*(2, 132) = 14.51, *p* < 0.0001, η^2^ = 0.18.

Planned comparisons were conducted contrasting decision times between the two distractor task groups for each vignette category. (All tests two-tailed.) When deontological principles were violated, participants took equally long to make decisions following the emotion rating task and the cognitive task; the same was true when deontological principles were not violated, all *t*’s < 1. Similarly, the type of distractor task did not differentially impact decision times for High Conflict, Moderate Conflict-No, and Moderate Conflict-Yes vignettes, *t*’s < 1. This rules out the possibility that differences in decision outcomes between the emotion and cognitive groups were due to differences in the amount of time spent on deliberation following completion of their respective tasks.

Collapsing across distractor task, planned comparisons replicated the results of Experiments 1 and 2: Participants once again took longer to make decisions about vignettes that did not violate deontological principles (*M* = 3.56) than vignettes that did (*M* = 3.30) *t*(189) = 2.07, *p* < 0.05.

Participants took longer to render judgments for high decisional conflict dilemmas (*M* = 3.98) than for moderate decisional conflict dilemmas, Mod-No = 3.13, *t*(89) = 5.11, *p* < 0.0001; Mod-Yes = 3.18, *t*(89) = 4.57, *p* < 0.0001. The moderate conflict dilemmas did not differ from each other, *t* < 1.

#### Summary of decisional conflict and deontological analyses

This pattern of results indicates that (a) dilemmas whose consensus and decision time profiles are consistent with high decisional conflict were strongly influenced by emotional state, (b) exposure to pleasant emotional stimuli decreased utilitarian judgments, (c) the impact of emotion did not depend on whether deontological violations occurred, and (d) the impact of emotion was not due to changes in the amount of time spent in decisional processing. These results suggest that reducing the stress involved in resolving decisional conflicts between early deontological and later utilitarian outcomes is responsible for shifting the balance in favor of deontological judgments. This pattern of results is inconsistent with the claim that prepotent negative emotional responses elicited by deontological violations must be overcome in order to render utilitarian judgments.

## Discussion

In these experiments, decisional conflict was defined in two ways, (a) as a conflict between utilitarian and deontological principles, and (b) as long decision times coupled with low inter-subject consensus. The results of Experiment 1 showed that when decision time was truncated, people rendered fewer utilitarian judgments for dilemmas that induce high decisional conflict when defined in either way. Experiment 2 replicated this effect and showed that when given more time, they were more likely to change their deontological judgments into utilitarian ones than vice versa. These results held regardless of whether a “yes” or “no” decision constituted a utilitarian judgment. This overall pattern of results is consistent with Greene’s (2007) dual-process model of moral judgment in which deontological judgments are the domain of a fast System 1 process while utilitarian judgments are handled by a slower, deliberative System 2. They also confirm the validity of personal force and intentional harm as important deontological principles in moral judgment.

Two findings were inconsistent with the predictions of the model. First, the longest decision times should obtain when rapid deontological judgments conflict with slower utilitarian judgments. Yet such conflicts were associated with rapid decision times. All dilemmas described the same utilitarian structure of harming few to save or help many. Yet people were found to quickly reject these actions if they also required intentionally harming someone through the use of personal force. When neither personal force nor intentional harm is involved, they took more time to reach a decision.

Second, the results of Experiment 3 were not consistent with the involvement of emotion in deontological judgments. According to the model, deontological violations elicit strong negative affect which biases the judgment outcome toward rejecting the proposed course of action described in the dilemma – contrary to utilitarian concerns. Reducing negative affect therefore should have increased the frequency of utilitarian judgments. Instead, the opposite obtained: Exposure to pleasant emotional stimuli was found to *decrease* utilitarian judgments for high decisional conflict dilemmas, regardless of whether the dilemmas involved deontological violations. This result suggests that the stress of decisional conflict is the crucial factor that interferes with deliberative reasoning, not the negative affect that is induced by deontological violations. This leaves open the possibility that deontological judgments may be described more as a rapid, heuristic process than an emotion-driven one.

Our results also show that, decisional conflict cannot be predicted on the basis of deontological violations as was done by Suter and Hertwig (2011) and others. Dilemmas whose decisional profiles reflected long decision times and low inter-subject consensus were not necessarily the same ones that involved deontological violations, nor were dilemmas that boast rapid times and high utilitarian consensus necessarily the same ones that were free of deontological violations. Other factors seem to be responsible for at least some of these decisional conflict profiles. For this reason, caution should be observed when defining dilemmas as “high” or “low” entirely theoretically, without taking empirical decisional profiles into account.

It should be noted that our results are not inconsistent with dual-process explanations of moral judgment in general. Our experiments were designed to test a specific dual-process model – the model proposed by Greene et al. (2001) and Greene (2007). According to that model, when deontological principles are violated, fast negative affect is evoked which must be overcome by slower deliberation. Our results are inconsistent with this aspect of the model: The presence or absence of violations of deontological principles was not predictive of differences in early, affect-based judgment (Experiments 1 and 2), and diminishing negative emotional affect led to more deontological judgments, not fewer (Experiment 3).

Most importantly, these results are relevant to teasing apart the separate contributions of intuition, emotion, and heuristic processes in moral judgment. Each of these typically yield rapid decisional outputs. Previous research suggested that utilitarian judgments may be decreased by inducing disgust (Schnall et al., 2008), stress (Youssef et al., 2012), or “elevation” (benevolence; Strohminger et al., 2011). Conversely, making light of deontological violations by making them seem funny increases utilitarian judgments (Valdesolo and DeSteno, 2006; Strohminger et al., 2011). Our results add to this literature by showing that exposure to pleasant emotional stimuli dramatically reduces utilitarian judgments, while inclusion of a cognitive task did not impact moral judgments. This suggests that the impact of the emotion task was not due to simple stress reduction. Instead, the type or quality of the emotion elicited appears crucial to moral judgment outcomes.

Our results are also inconsistent with the predictions of theories which treat emotions as epiphenomena which play no substantive role in moral judgment (Rawls, 1971; Cushman et al., 2006; Hauser et al., 2006). Haidt (2001, 2007) has proposed that moral judgments are primarily fast, intuition-based judgments, and reasoning occurs only later to justify a judgment already made. Our results show that moral judgments do indeed occur quite rapidly, but the results of Experiment 2 show that these rapid judgments are frequently reversed upon more reflection. The most accurate interpretation of our results appears to be the moral judgment involves both fast and slow decisional processes, and that early emotional responses play an important role that is not yet well understood.

Our results seem to indicate that person-based considerations often weigh more heavily in moral judgment than principle-based considerations. What seems to be most influential is victim characteristics, as seen in Table 2. Of the vignettes logging fewer than 65% utilitarian decisions, almost all of the victims belonged to a vulnerable class (i.e., child, patient, injured innocent) or had other characteristics that would elicit a compassion response (i.e., fat man, fellow hostage). Of the eight vignettes that logged greater than 65% utilitarian decisions, five had characteristics that would lessen compassion because danger or risk is part of their position (i.e., terrorist’s son, combatants, workmen, soldier, and fellow passenger) and two did not involve fatal harm. (The primary difference between “fellow hostage” and “fellow passenger” is that the former had no choice in becoming a hostage while the latter chose to enter the lifeboat.) This interpretation is consistent with other studies that have reported victim characteristics to weigh in moral judgment. For example, Cikara et al. (2010) found that intergroup biases and stereotypes weigh heavily on neural systems implicated in moral decision-making.

**Table 2:** **Victim characteristics of vignettes ranked according to % utilitarian decisions**.

Vignette	% Utilitarian	Victim
Hard times	3	Child
Transplant	5	Patient
Smother for dollars	8	Patient
Footbridge	15	Fat man
Modified safari	28	Fellow hostage
Sacrifice	28	Child
Fumes w/friends	36	Injured friend
Vitamins	38	Patient
Crying baby	40	Child
Sophie’s choice	41	Child
Cancer toddler	52	Child
Standard fumes	62	Injured innocent
Donation	67	n/a
Modified lifeboat	67	Fellow passenger
Lawrence of Arabia	68	Combatants
Vaccine test	68	Patients
Vaccine policy	75	n/a
Standard trolley	80	Workman
Submarine	80	Soldier
Modified bomb	85	Terrorist’s son

From this viewpoint, factors that elicit greater compassion for the victim or put the decision-maker in an emotional state that is conducive to compassion will decrease their willingness to impose harmful consequences on the victim, hence decreasing the likelihood of a utilitarian response. Our data indicate that this person-assessment process takes place rapidly in System 1, and results in affective responses that are primarily expressed in terms of degree of compassion for the victim. Harms to a vulnerable person are seen as a greater harm than harms to those less vulnerable. This is a utilitarian concern. If correct, then the identification of deontological judgments with a rapid, affective response and utilitarian judgments with a slower, deliberative response is not warranted. Instead, both deontological and utilitarian heuristics may be part of the System 1 process. Fast responses to these vignettes can obtain simply by applying a extremely simple “greater good” heuristic (i.e., always choose that action that maximizes the number of lives saved, regardless of victim characteristics), or by applying a deontological heuristic (e.g., never use a person as a means to an end). The deliberative aspect of a dual-process account involves the careful weighing of the outcomes of these heuristics, particularly when a conflict among the outputs occurs.

We tested an influential theory that explains moral judgment as the outcome of parallel rapid affective and slower deliberative processes. People were required to make decisions about moral dilemmas that involved harming some in order to save many. These dilemmas were selected on the basis of previous research to vary in terms of the gravity of the harm and hence the degree of affective response elicited. We found that restricting decision time (in order to limit time available for deliberation and increase task stress) had little impact on judgments that induced little decisional conflict, but significantly decreased the proportion of decisions that were consistent with deliberative aggregate benefit analyses for dilemmas that induced high conflict. We also found that requiring people to rate the emotional pleasantness of visual stimuli in a distractor task similarly decreased the proportion of utilitarian-type moral judgments. These results are consistent with the prediction that truncating decision time shifts decision profiles in favor of weighting affective features of the dilemma more heavily than their utilitarian structures.

Our findings are directly relevant to three other papers published in this volume. The work reported by Henderson et al. (2012) indicates that exposure to moderate, controllable stress benefits performance, but exposure to uncontrollable stress or having a more extreme response to stress tends to harm performance. The analysis provided by Kanske (2012) suggested that stress can be leveraged in order to benefit performance. But we found induction of even moderate stress through restricting decision time had a pronounced affect on moral judgments. Finally, Trübutschek and Egner (2012) demonstrate that previous reports of emotion-modulated trial–transition effects are likely attributable to the effects of emotion on cognitive control processes. Similarly, our results indicate that affective processes can strongly impact decisional profiles in predictable ways.

## Conflict of Interest Statement

The authors declare that the research was conducted in the absence of any commercial or financial relationships that could be construed as a potential conflict of interest.
